# {Bis[2-(diphenyl­phosphino)eth­yl]phenyl­phosphine-κ^3^ *P*,*P*′,*P*′′}chloridopalladium(II) hexa­fluoridophosphate

**DOI:** 10.1107/S1600536809022417

**Published:** 2009-06-20

**Authors:** Paul R. Vorce, Susie M. Miller, Monte L. Helm

**Affiliations:** a Chemistry Department, Fort Lewis College, 1000 Rim Drive, Durango, CO 81301, USA; b Chemistry Department, Colorado State University, Fort Collins, CO 80523, USA

## Abstract

In the title compound, [PdCl(C_34_H_33_P_3_)]PF_6_, the Pd^II^ atom adopts a distorted PdP_3_Cl square-planar geometry arising from the *P*,*P*′,*P*′′-tridentate triphos ligand and a chloride ion.

## Related literature

For the synthesis, see: King *et al.* (1971 ▶). The corresponding complex with a Pt^II^ metal center is concurently published (Heston *et al.*, 2009 ▶). The corresponding Pd^II^ complex has been previously reported as a trifluoro­methane­sulfonate salt (Müller *et al.*, 2000 ▶). The corresponding complexes with both Pt^II^ and Pd^II^ have been previously reported as chloride and diphenyl­tetra­chlorido­stannate(IV) salts (Sevillano *et al.*, 1999*a* ▶; Garcia-Seijo *et al.*, 2001 ▶; Housecroft *et al.*, 1990 ▶). For other group 10–triphos complexes, see: Sevillano *et al.* (1999*b* ▶); Fernadez *et al.* (2005); Aizawa *et al.* (2002 ▶); Bertinsson *et al.* (1983 ▶); Autissier *et al.* (2005 ▶); Fernandez *et al.* (2005 ▶).
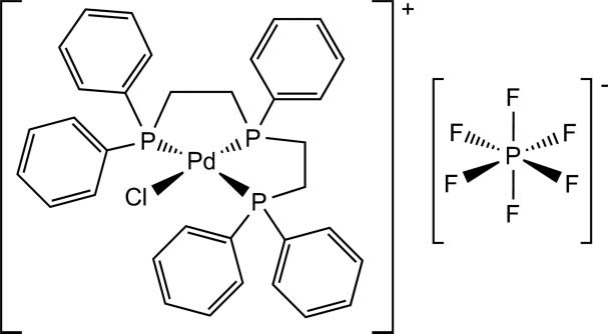


## Experimental

### 

#### Crystal data

[PdCl(C_34_H_33_P_3_)]PF_6_
*M* *_r_* = 821.33
Triclinic, 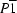

*a* = 11.2465 (5) Å
*b* = 11.8182 (5) Å
*c* = 15.5093 (7) Å
α = 69.029 (2)°
β = 70.439 (2)°
γ = 69.697 (2)°
*V* = 1752.27 (13) Å^3^
*Z* = 2
Mo *K*α radiation
μ = 0.84 mm^−1^
*T* = 100 K
0.47 × 0.15 × 0.10 mm

#### Data collection

Bruker APEXII CCD diffractometer
Absorption correction: multi-scan (*SADABS*; Bruker, 2000 ▶) *T*_min_ = 0.693, *T*_max_ = 0.917
48576 measured reflections
13341 independent reflections
10917 reflections with *I* > 2σ(*I*)
*R*_int_ = 0.030

#### Refinement

*R*[*F* ^2^ > 2σ(*F* ^2^)] = 0.031
*wR*(*F* ^2^) = 0.067
*S* = 1.03
13341 reflections
415 parameters
H-atom parameters constrained
Δρ_max_ = 0.71 e Å^−3^
Δρ_min_ = −0.67 e Å^−3^

### 

Data collection: *APEX2* (Bruker, 2006 ▶); cell refinement: *SAINT* (Bruker, 2006 ▶); data reduction: *SAINT*; program(s) used to solve structure: *SHELXS97* (Sheldrick, 2008 ▶); program(s) used to refine structure: *SHELXL97* (Sheldrick, 2008 ▶); molecular graphics: *SHELXTL* (Sheldrick, 2008 ▶); software used to prepare material for publication: *SHELXTL*.

## Supplementary Material

Crystal structure: contains datablocks global, I. DOI: 10.1107/S1600536809022417/hb2992sup1.cif

Structure factors: contains datablocks I. DOI: 10.1107/S1600536809022417/hb2992Isup2.hkl

Additional supplementary materials:  crystallographic information; 3D view; checkCIF report

**Fig. 1.**
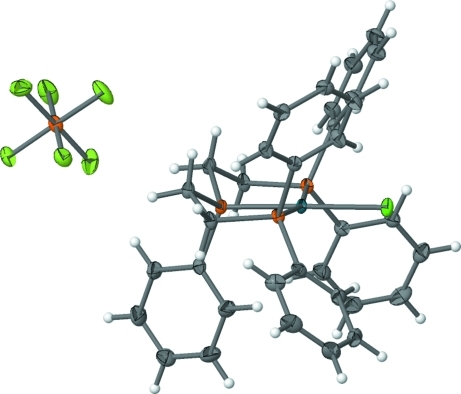
The molecular structure of (I) with 50% probability displacement ellipsoids.

Enhanced figure: interactive version of Fig. 1

## Figures and Tables

**Table 1:** Selected bond lengths (Å)

Pd1—P1	2.2176 (4)
Pd1—P2	2.3178 (4)
Pd1—P3	2.3329 (4)
Pd1—Cl1	2.3441 (4)

**Fig. 2.**
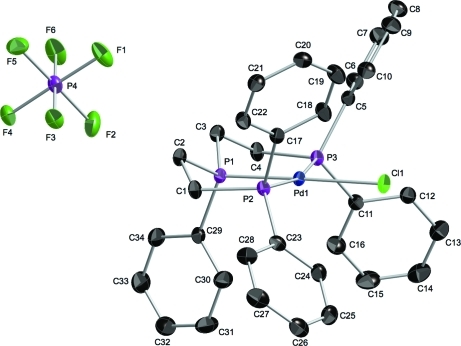
The molecular structure of (I), with atom labels and 50% probability displacement ellipsoids. Hydrogen atoms are omitted for clarity.

## supplementary crystallographic information

### Comment

The crystal structure of the title compound, (I), consists of a [Pd(triphos)Cl]^+^ cation and PF_6_^-^ anion (Fig. 1). The cation shows a distorted square planar geometry around the metal center and the charge is balanced by a non-coordinating PF_6_^-^ anion.

### Experimental

The title compound was prepared by a previously reported proceedure (King, *et al.*, 1971). Colourless rods of (I) were grown by slow solvent evaporation of a saturated dichloromethane solution.

### Refinement

All H atoms were placed in calculated positions (C—H = 0.93–0.97Å) and refined as riding with *U*_iso_(H) = 1.2*U*_eq_(C).

### Crystal data

**Table:**

[PdCl(C_34_H_33_P_3_)]PF_6_	*Z* = 2
*M**_r_* = 821.33	*F*(000) = 828
Triclinic, *P*1	*D*_x_ = 1.557 Mg m^−^^3^
Hall symbol: -P 1	Melting point: not measured K
*a* = 11.2465 (5) Å	Mo *K*α radiation, λ = 0.71073 Å
*b* = 11.8182 (5) Å	Cell parameters from 9890 reflections
*c* = 15.5093 (7) Å	θ = 2.7–33.3°
α = 69.029 (2)°	µ = 0.84 mm^−^^1^
β = 70.439 (2)°	*T* = 100 K
γ = 69.697 (2)°	Rod, colourless
*V* = 1752.27 (13) Å^3^	0.47 × 0.15 × 0.10 mm

### Data collection

**Table:**

Bruker APEXII CCD diffractometer	13341 independent reflections
Radiation source: fine-focus sealed tube	10917 reflections with *I* > 2σ(*I*)
graphite	*R*_int_ = 0.030
φ and ω scans	θ_max_ = 33.4°, θ_min_ = 1.9°
Absorption correction: multi-scan (*SADABS*; Bruker, 2000)	*h* = −16→17
*T*_min_ = 0.693, *T*_max_ = 0.917	*k* = −18→18
48576 measured reflections	*l* = −23→21

### Refinement

**Table:**

Refinement on *F*^2^	Primary atom site location: structure-invariant direct methods
Least-squares matrix: full	Secondary atom site location: difference Fourier map
*R*[*F*^2^ > 2σ(*F*^2^)] = 0.031	Hydrogen site location: inferred from neighbouring sites
*wR*(*F*^2^) = 0.067	H-atom parameters constrained
*S* = 1.03	*w* = 1/[σ^2^(*F*_o_^2^) + (0.0249*P*)^2^ + 0.7353*P*] where *P* = (*F*_o_^2^ + 2*F*_c_^2^)/3
13341 reflections	(Δ/σ)_max_ = 0.002
415 parameters	Δρ_max_ = 0.71 e Å^−^^3^
0 restraints	Δρ_min_ = −0.67 e Å^−^^3^

### Special details

**Table:**

Geometry. All e.s.d.'s (except the e.s.d. in the dihedral angle between two l.s. planes) are estimated using the full covariance matrix. The cell e.s.d.'s are taken into account individually in the estimation of e.s.d.'s in distances, angles and torsion angles; correlations between e.s.d.'s in cell parameters are only used when they are defined by crystal symmetry. An approximate (isotropic) treatment of cell e.s.d.'s is used for estimating e.s.d.'s involving l.s. planes.
Refinement. Refinement of *F*^2^ against ALL reflections. The weighted *R*-factor *wR* and goodness of fit *S* are based on *F*^2^, conventional *R*-factors *R* are based on *F*, with *F* set to zero for negative *F*^2^. The threshold expression of *F*^2^ > σ(*F*^2^) is used only for calculating *R*-factors(gt) *etc*. and is not relevant to the choice of reflections for refinement. *R*-factors based on *F*^2^ are statistically about twice as large as those based on *F*, and *R*- factors based on ALL data will be even larger.

### Fractional atomic coordinates and isotropic or equivalent isotropic displacement parameters (Å^2^)

**Table:**

	*x*	*y*	*z*	*U*_iso_*/*U*_eq_	
Pd1	0.543690 (10)	0.544468 (10)	0.728992 (7)	0.01221 (3)	
Cl1	0.66556 (3)	0.69224 (3)	0.64082 (3)	0.01911 (7)	
P1	0.41725 (3)	0.41349 (3)	0.80705 (3)	0.01332 (7)	
P2	0.34703 (3)	0.69724 (3)	0.73350 (3)	0.01374 (7)	
P3	0.71382 (3)	0.36524 (3)	0.75901 (3)	0.01384 (7)	
C1	0.63963 (14)	0.23211 (14)	0.82230 (11)	0.0178 (3)	
H1A	0.6334	0.1964	0.7756	0.021*	
H1B	0.6956	0.1658	0.8631	0.021*	
C2	0.50294 (14)	0.27458 (14)	0.88395 (11)	0.0177 (3)	
H2A	0.5089	0.2948	0.9388	0.021*	
H2B	0.4566	0.2077	0.9082	0.021*	
C3	0.26481 (14)	0.49798 (14)	0.87132 (11)	0.0182 (3)	
H3A	0.2009	0.4465	0.9003	0.022*	
H3B	0.2800	0.5191	0.9224	0.022*	
C4	0.21432 (13)	0.61759 (14)	0.79763 (11)	0.0184 (3)	
H4A	0.1379	0.6736	0.8297	0.022*	
H4B	0.1869	0.5962	0.7523	0.022*	
C5	0.32177 (14)	0.80673 (14)	0.79914 (10)	0.0156 (3)	
C6	0.19906 (14)	0.89177 (15)	0.81373 (11)	0.0197 (3)	
H6A	0.1315	0.8893	0.7911	0.024*	
C7	0.17601 (16)	0.97952 (16)	0.86117 (12)	0.0242 (3)	
H7A	0.0925	1.0370	0.8713	0.029*	
C8	0.27491 (17)	0.98361 (16)	0.89391 (12)	0.0261 (4)	
H8A	0.2591	1.0444	0.9260	0.031*	
C9	0.39634 (16)	0.89942 (17)	0.87998 (12)	0.0250 (3)	
H9A	0.4634	0.9022	0.9029	0.030*	
C10	0.42046 (15)	0.81067 (15)	0.83243 (11)	0.0207 (3)	
H10A	0.5039	0.7531	0.8228	0.025*	
C11	0.31785 (13)	0.79486 (14)	0.61900 (10)	0.0158 (3)	
C12	0.35593 (15)	0.90719 (15)	0.57975 (11)	0.0193 (3)	
H12A	0.3897	0.9338	0.6156	0.023*	
C13	0.34469 (16)	0.97972 (16)	0.48887 (12)	0.0259 (4)	
H13A	0.3710	1.0557	0.4625	0.031*	
C14	0.29516 (17)	0.94151 (18)	0.43648 (12)	0.0305 (4)	
H14A	0.2872	0.9913	0.3742	0.037*	
C15	0.25735 (19)	0.8311 (2)	0.47485 (13)	0.0337 (4)	
H15A	0.2228	0.8055	0.4389	0.040*	
C16	0.26919 (17)	0.75693 (18)	0.56544 (13)	0.0271 (4)	
H16A	0.2441	0.6803	0.5909	0.033*	
C17	0.79995 (13)	0.37155 (14)	0.83702 (10)	0.0156 (3)	
C18	0.81699 (16)	0.48626 (15)	0.82975 (12)	0.0222 (3)	
H18A	0.7779	0.5608	0.7880	0.027*	
C19	0.89105 (16)	0.49210 (16)	0.88346 (13)	0.0245 (3)	
H19A	0.9026	0.5705	0.8781	0.029*	
C20	0.94768 (14)	0.38410 (15)	0.94455 (11)	0.0200 (3)	
H20A	0.9981	0.3883	0.9812	0.024*	
C21	0.93115 (15)	0.26977 (16)	0.95254 (11)	0.0211 (3)	
H21A	0.9708	0.1956	0.9943	0.025*	
C22	0.85680 (15)	0.26298 (15)	0.89975 (11)	0.0201 (3)	
H22A	0.8446	0.1845	0.9062	0.024*	
C23	0.84173 (13)	0.31713 (15)	0.66107 (11)	0.0173 (3)	
C24	0.85893 (17)	0.40133 (16)	0.57143 (12)	0.0253 (3)	
H24A	0.8039	0.4843	0.5612	0.030*	
C25	0.95762 (18)	0.36365 (18)	0.49618 (13)	0.0327 (4)	
H25A	0.9700	0.4211	0.4347	0.039*	
C26	1.03721 (16)	0.24272 (18)	0.51127 (14)	0.0304 (4)	
H26A	1.1042	0.2173	0.4600	0.037*	
C27	1.01997 (16)	0.15849 (18)	0.60052 (13)	0.0286 (4)	
H27A	1.0747	0.0754	0.6102	0.034*	
C28	0.92312 (15)	0.19491 (16)	0.67579 (12)	0.0231 (3)	
H28A	0.9119	0.1372	0.7372	0.028*	
C29	0.37076 (14)	0.36381 (14)	0.72824 (11)	0.0170 (3)	
C30	0.43949 (16)	0.37635 (18)	0.63357 (12)	0.0264 (4)	
H30A	0.5137	0.4095	0.6100	0.032*	
C31	0.40025 (19)	0.3407 (2)	0.57341 (13)	0.0337 (4)	
H31A	0.4483	0.3484	0.5091	0.040*	
C32	0.29116 (19)	0.29404 (19)	0.60692 (13)	0.0313 (4)	
H32A	0.2642	0.2702	0.5655	0.038*	
C33	0.2214 (2)	0.2821 (2)	0.70068 (14)	0.0359 (5)	
H33A	0.1459	0.2511	0.7233	0.043*	
C34	0.26129 (17)	0.31525 (18)	0.76138 (12)	0.0289 (4)	
H34A	0.2142	0.3050	0.8260	0.035*	
P4	0.21871 (4)	0.13320 (4)	1.11479 (3)	0.01765 (8)	
F1	0.14268 (9)	0.04474 (9)	1.10733 (7)	0.0233 (2)	
F2	0.23404 (11)	0.04420 (10)	1.21717 (7)	0.0334 (2)	
F3	0.35426 (8)	0.05090 (9)	1.06650 (7)	0.0241 (2)	
F4	0.29314 (10)	0.22256 (10)	1.12031 (9)	0.0334 (3)	
F5	0.20332 (10)	0.22185 (10)	1.01037 (8)	0.0341 (3)	
F6	0.08134 (9)	0.21610 (10)	1.16043 (9)	0.0359 (3)	

### Atomic displacement parameters (Å^2^)

**Table:**

	*U*^11^	*U*^22^	*U*^33^	*U*^12^	*U*^13^	*U*^23^
Pd1	0.01063 (5)	0.00951 (5)	0.01375 (5)	−0.00185 (3)	−0.00232 (3)	−0.00135 (4)
Cl1	0.01759 (15)	0.01486 (17)	0.02136 (17)	−0.00688 (13)	−0.00301 (13)	0.00013 (13)
P1	0.01253 (15)	0.01079 (17)	0.01399 (16)	−0.00290 (13)	−0.00164 (12)	−0.00189 (13)
P2	0.01197 (15)	0.01054 (17)	0.01684 (17)	−0.00140 (12)	−0.00385 (13)	−0.00272 (13)
P3	0.01189 (15)	0.01076 (17)	0.01771 (17)	−0.00135 (13)	−0.00379 (13)	−0.00387 (14)
C1	0.0156 (6)	0.0118 (7)	0.0239 (7)	−0.0031 (5)	−0.0050 (5)	−0.0026 (6)
C2	0.0174 (6)	0.0135 (7)	0.0188 (7)	−0.0043 (5)	−0.0045 (5)	−0.0001 (5)
C3	0.0151 (6)	0.0159 (7)	0.0193 (7)	−0.0039 (5)	0.0005 (5)	−0.0044 (6)
C4	0.0133 (6)	0.0129 (7)	0.0255 (7)	−0.0031 (5)	−0.0019 (5)	−0.0041 (6)
C5	0.0170 (6)	0.0122 (7)	0.0156 (6)	−0.0024 (5)	−0.0044 (5)	−0.0025 (5)
C6	0.0180 (6)	0.0178 (7)	0.0232 (7)	−0.0020 (6)	−0.0060 (6)	−0.0072 (6)
C7	0.0242 (7)	0.0193 (8)	0.0272 (8)	−0.0009 (6)	−0.0040 (6)	−0.0107 (7)
C8	0.0322 (8)	0.0220 (8)	0.0270 (8)	−0.0052 (7)	−0.0079 (7)	−0.0112 (7)
C9	0.0268 (8)	0.0274 (9)	0.0263 (8)	−0.0076 (7)	−0.0105 (6)	−0.0092 (7)
C10	0.0198 (7)	0.0208 (8)	0.0218 (7)	−0.0023 (6)	−0.0081 (6)	−0.0059 (6)
C11	0.0137 (6)	0.0138 (7)	0.0189 (7)	−0.0003 (5)	−0.0066 (5)	−0.0040 (5)
C12	0.0211 (7)	0.0158 (7)	0.0201 (7)	−0.0033 (6)	−0.0065 (6)	−0.0038 (6)
C13	0.0264 (8)	0.0199 (8)	0.0229 (8)	−0.0027 (6)	−0.0062 (6)	0.0010 (6)
C14	0.0279 (8)	0.0363 (10)	0.0194 (8)	−0.0009 (7)	−0.0116 (7)	−0.0002 (7)
C15	0.0347 (9)	0.0471 (12)	0.0274 (9)	−0.0119 (9)	−0.0166 (8)	−0.0099 (9)
C16	0.0310 (8)	0.0294 (9)	0.0278 (9)	−0.0122 (7)	−0.0122 (7)	−0.0067 (7)
C17	0.0130 (6)	0.0133 (7)	0.0179 (7)	−0.0010 (5)	−0.0030 (5)	−0.0043 (5)
C18	0.0245 (7)	0.0138 (7)	0.0293 (8)	−0.0023 (6)	−0.0129 (6)	−0.0037 (6)
C19	0.0258 (8)	0.0186 (8)	0.0347 (9)	−0.0047 (6)	−0.0128 (7)	−0.0094 (7)
C20	0.0162 (6)	0.0237 (8)	0.0213 (7)	−0.0029 (6)	−0.0053 (5)	−0.0091 (6)
C21	0.0212 (7)	0.0205 (8)	0.0199 (7)	−0.0038 (6)	−0.0082 (6)	−0.0022 (6)
C22	0.0227 (7)	0.0145 (7)	0.0229 (7)	−0.0045 (6)	−0.0080 (6)	−0.0029 (6)
C23	0.0136 (6)	0.0197 (7)	0.0224 (7)	−0.0052 (5)	−0.0033 (5)	−0.0100 (6)
C24	0.0296 (8)	0.0197 (8)	0.0252 (8)	−0.0102 (7)	0.0011 (6)	−0.0080 (7)
C25	0.0392 (10)	0.0307 (10)	0.0258 (9)	−0.0207 (8)	0.0096 (7)	−0.0098 (8)
C26	0.0206 (7)	0.0380 (11)	0.0365 (10)	−0.0127 (7)	0.0074 (7)	−0.0228 (8)
C27	0.0181 (7)	0.0313 (10)	0.0370 (10)	0.0007 (7)	−0.0048 (7)	−0.0188 (8)
C28	0.0186 (7)	0.0244 (8)	0.0251 (8)	0.0008 (6)	−0.0076 (6)	−0.0093 (7)
C29	0.0179 (6)	0.0135 (7)	0.0188 (7)	−0.0043 (5)	−0.0056 (5)	−0.0022 (5)
C30	0.0228 (7)	0.0368 (10)	0.0224 (8)	−0.0134 (7)	−0.0001 (6)	−0.0107 (7)
C31	0.0355 (9)	0.0511 (13)	0.0214 (8)	−0.0196 (9)	−0.0027 (7)	−0.0136 (8)
C32	0.0380 (10)	0.0370 (11)	0.0283 (9)	−0.0175 (8)	−0.0132 (7)	−0.0071 (8)
C33	0.0402 (10)	0.0468 (12)	0.0322 (10)	−0.0302 (9)	−0.0076 (8)	−0.0065 (9)
C34	0.0325 (9)	0.0381 (11)	0.0220 (8)	−0.0231 (8)	−0.0022 (7)	−0.0055 (7)
P4	0.01521 (16)	0.01243 (18)	0.0248 (2)	−0.00147 (14)	−0.00703 (14)	−0.00449 (15)
F1	0.0217 (4)	0.0182 (5)	0.0329 (5)	−0.0076 (4)	−0.0095 (4)	−0.0054 (4)
F2	0.0469 (6)	0.0305 (6)	0.0226 (5)	−0.0058 (5)	−0.0139 (5)	−0.0061 (4)
F3	0.0169 (4)	0.0213 (5)	0.0338 (5)	−0.0021 (4)	−0.0041 (4)	−0.0118 (4)
F4	0.0237 (5)	0.0232 (5)	0.0630 (8)	−0.0036 (4)	−0.0147 (5)	−0.0214 (5)
F5	0.0362 (6)	0.0240 (5)	0.0363 (6)	−0.0103 (5)	−0.0185 (5)	0.0098 (5)
F6	0.0185 (4)	0.0226 (5)	0.0633 (8)	0.0007 (4)	−0.0014 (5)	−0.0216 (5)

### Geometric parameters (Å, °)

**Table:**

Pd1—P1	2.2176 (4)	C15—C16	1.387 (3)
Pd1—P2	2.3178 (4)	C15—H15A	0.9500
Pd1—P3	2.3329 (4)	C16—H16A	0.9500
Pd1—Cl1	2.3441 (4)	C17—C18	1.394 (2)
P1—C29	1.8073 (16)	C17—C22	1.398 (2)
P1—C3	1.8193 (14)	C18—C19	1.392 (2)
P1—C2	1.8211 (15)	C18—H18A	0.9500
P2—C11	1.8037 (15)	C19—C20	1.381 (2)
P2—C5	1.8126 (15)	C19—H19A	0.9500
P2—C4	1.8476 (15)	C20—C21	1.384 (2)
P3—C23	1.8132 (15)	C20—H20A	0.9500
P3—C17	1.8185 (16)	C21—C22	1.389 (2)
P3—C1	1.8418 (15)	C21—H21A	0.9500
C1—C2	1.534 (2)	C22—H22A	0.9500
C1—H1A	0.9900	C23—C24	1.387 (2)
C1—H1B	0.9900	C23—C28	1.401 (2)
C2—H2A	0.9900	C24—C25	1.398 (2)
C2—H2B	0.9900	C24—H24A	0.9500
C3—C4	1.532 (2)	C25—C26	1.383 (3)
C3—H3A	0.9900	C25—H25A	0.9500
C3—H3B	0.9900	C26—C27	1.383 (3)
C4—H4A	0.9900	C26—H26A	0.9500
C4—H4B	0.9900	C27—C28	1.385 (2)
C5—C10	1.392 (2)	C27—H27A	0.9500
C5—C6	1.399 (2)	C28—H28A	0.9500
C6—C7	1.384 (2)	C29—C30	1.393 (2)
C6—H6A	0.9500	C29—C34	1.402 (2)
C7—C8	1.389 (2)	C30—C31	1.388 (3)
C7—H7A	0.9500	C30—H30A	0.9500
C8—C9	1.384 (2)	C31—C32	1.385 (3)
C8—H8A	0.9500	C31—H31A	0.9500
C9—C10	1.393 (2)	C32—C33	1.384 (3)
C9—H9A	0.9500	C32—H32A	0.9500
C10—H10A	0.9500	C33—C34	1.382 (3)
C11—C16	1.390 (2)	C33—H33A	0.9500
C11—C12	1.399 (2)	C34—H34A	0.9500
C12—C13	1.384 (2)	P4—F1	1.6158 (10)
C12—H12A	0.9500	P4—F2	1.5872 (11)
C13—C14	1.384 (3)	P4—F3	1.5997 (10)
C13—H13A	0.9500	P4—F4	1.5967 (11)
C14—C15	1.378 (3)	P4—F6	1.6005 (10)
C14—H14A	0.9500	P4—F5	1.6117 (11)
			
P1—Pd1—P2	83.905 (14)	C14—C15—H15A	120.1
P1—Pd1—P3	84.262 (14)	C16—C15—H15A	120.1
P2—Pd1—P3	166.221 (13)	C15—C16—C11	119.90 (17)
P1—Pd1—Cl1	175.897 (14)	C15—C16—H16A	120.1
P2—Pd1—Cl1	92.644 (14)	C11—C16—H16A	120.1
P3—Pd1—Cl1	99.430 (14)	C18—C17—C22	119.14 (15)
C29—P1—C3	105.15 (7)	C18—C17—P3	119.17 (12)
C29—P1—C2	108.28 (7)	C22—C17—P3	121.56 (12)
C3—P1—C2	113.38 (7)	C19—C18—C17	120.29 (15)
C29—P1—Pd1	112.53 (5)	C19—C18—H18A	120.1
C3—P1—Pd1	107.87 (5)	C17—C18—H18A	120.1
C2—P1—Pd1	109.64 (5)	C20—C19—C18	120.05 (15)
C11—P2—C5	104.67 (7)	C20—C19—H19A	120.1
C11—P2—C4	108.65 (7)	C18—C19—H19A	120.1
C5—P2—C4	104.60 (7)	C19—C20—C21	120.16 (15)
C11—P2—Pd1	115.24 (5)	C19—C20—H20A	120.1
C5—P2—Pd1	115.29 (5)	C21—C20—H20A	120.1
C4—P2—Pd1	107.76 (5)	C20—C21—C22	120.26 (15)
C23—P3—C17	104.71 (7)	C20—C21—H21A	120.1
C23—P3—C1	105.01 (7)	C22—C21—H21A	120.1
C17—P3—C1	106.71 (7)	C21—C22—C17	120.09 (15)
C23—P3—Pd1	120.12 (5)	C21—C22—H22A	120.1
C17—P3—Pd1	112.42 (5)	C17—C22—H22A	120.1
C1—P3—Pd1	106.97 (5)	C24—C23—C28	119.94 (14)
C1^i^—C2—H2A	109.7	C24—C23—P3	119.98 (12)
P1—C2—H2A	111.0	C28—C23—P3	120.07 (12)
C1^i^—C2—H2B	109.7	C23—C24—C25	119.72 (16)
P1—C2—H2B	111.0	C23—C24—H24A	120.1
C2—C1—P3	110.54 (10)	C25—C24—H24A	120.1
C2^i^—C1—H1A	109.7	C26—C25—C24	119.95 (17)
P3—C1—H1A	109.7	C26—C25—H25A	120.1
C2^i^—C1—H1B	109.7	C24—C25—H25A	120.1
P3—C1—H1B	109.7	C25—C26—C27	120.43 (15)
C1—C2—P1	106.09 (10)	C25—C26—H26A	120.1
C4—C3—P1	106.05 (10)	C27—C26—H26A	120.1
C4^i^—C3—H3A	109.7	C26—C27—C28	120.18 (17)
P1—C3—H3A	111.0	C26—C27—H27A	120.1
C4^i^—C3—H3B	109.7	C28—C27—H27A	120.1
P1—C3—H3B	111.0	C27—C28—C23	119.77 (16)
C3—C4—P2	108.46 (10)	C27—C28—H28A	120.1
C3^i^—C4—H4A	109.7	C23—C28—H28A	120.1
P2—C4—H4A	111.0	C30—C29—C34	118.84 (15)
C3^i^—C4—H4B	109.7	C30—C29—P1	121.20 (12)
P3—C4—H4B	103.0	C34—C29—P1	119.94 (12)
C10—C5—C6	119.82 (14)	C31—C30—C29	120.39 (15)
C10—C5—P2	122.34 (11)	C31—C30—H30A	120.1
C6—C5—P2	117.82 (12)	C29—C30—H30A	120.1
C7—C6—C5	120.02 (15)	C32—C31—C30	120.12 (16)
C7—C6—H6A	120.1	C32—C31—H31A	120.1
C5—C6—H6A	120.1	C30—C31—H31A	120.1
C6—C7—C8	120.02 (15)	C33—C32—C31	120.01 (17)
C6—C7—H7A	120.1	C33—C32—H32A	120.1
C8—C7—H7A	120.1	C31—C32—H32A	120.1
C9—C8—C7	120.21 (16)	C34—C33—C32	120.17 (16)
C9—C8—H8A	120.1	C34—C33—H33A	120.1
C7—C8—H8A	120.1	C32—C33—H33A	120.1
C8—C9—C10	120.21 (16)	C33—C34—C29	120.46 (16)
C8—C9—H9A	120.1	C33—C34—H34A	120.1
C10—C9—H9A	120.1	C29—C34—H34A	120.1
C5—C10—C9	119.72 (15)	F2—P4—F4	91.25 (6)
C5—C10—H10A	120.1	F2—P4—F3	90.49 (6)
C9—C10—H10A	120.1	F4—P4—F3	90.41 (5)
C16—C11—C12	119.14 (14)	F2—P4—F6	90.92 (6)
C16—C11—P2	121.99 (12)	F4—P4—F6	90.63 (6)
C12—C11—P2	118.63 (12)	F3—P4—F6	178.24 (6)
C13—C12—C11	120.33 (15)	F2—P4—F5	179.18 (6)
C13—C12—H12A	120.1	F4—P4—F5	89.44 (6)
C11—C12—H12A	120.1	F3—P4—F5	89.06 (6)
C14—C13—C12	120.02 (16)	F6—P4—F5	89.53 (6)
C14—C13—H13A	120.1	F2—P4—F1	89.86 (6)
C12—C13—H13A	120.1	F4—P4—F1	178.88 (6)
C15—C14—C13	119.87 (16)	F3—P4—F1	89.74 (5)
C15—C14—H14A	120.1	F6—P4—F1	89.19 (5)
C13—C14—H14A	120.1	F5—P4—F1	89.45 (6)
C14—C15—C16	120.72 (17)		

Symmetry codes: (i) .

## References

[bb1] Aizawa, S., Sone, Y., Kawamoto, T., Yamada, S. & Nakamura, M. (2002). *Inorg. Chim. Acta*, **338**, 235–239.

[bb2] Autissier, V., Brockman, E., Clegg, W., Harrington, R. W. & Henderson, R. A. (2005). *J. Organomet. Chem.* **690**, 1763–1771.

[bb3] Bertinsson, G.-I. (1983). *Acta Cryst.* C**39**, 563–567.

[bb15] Bruker (2000). *SADABS* Bruker AXS Inc., Madison, Wisconsin, USA.

[bb5] Bruker (2006). *APEX2* and *SAINT* Bruker AXS Inc., Madison, Wisconsin, USA.

[bb6] Fernandez, D., Garcia-Seijo, M. I., Sevillano, P., Castineiras, A. & Garcia-Fernandez, M. E. (2005). *Inorg. Chim. Acta*, **358**, 2575–2584.

[bb7] Garcia-Seijo, M. I., Castineiras, A., Mahieu, B., Janosi, L., Berente, Z., Kollar, L. & Garcia-Fernandez, M. E. (2001). *Polyhedron*, **20**, 855–868.

[bb8] Heston, S. A., Noll, B. C. & Helm, M. L. (2009). *Acta Cryst.* E**65**, m793. 10.1107/S1600536809022405 PMC2969258 21582720

[bb9] Housecroft, C. E., Shaykh, B. A. M., Rheingold, A. L. & Haggerty, B. S. (1990). *Acta Cryst.* C**46**, 1549–1551.

[bb10] King, R. B., Kapoor, P. N. & Kapoor, R. N. (1971). *Inorg. Chem.* **10**, 1841–1850.

[bb11] Müller, T. E., Grosche, M., Herdtweck, E., Pleier, A.-K., Walter, E. & Yaw-Kai, Y. (2000). *Organometallics*, **19**, 170–183.

[bb12] Sevillano, P., Habtemariam, A., Parsons, S., Castineiras, A., Garcia, M. E. & Sadler, P. J. (1999*a*). *J. Chem. Soc. Dalton Trans.* pp. 2861–2870.

[bb13] Sevillano, P., Habtemariam, A., Parsons, S., Castineiras, A., Garcia, M. E. & Sadler, P. J. (1999*b*). *Polyhedron*, **18**, 383–389.

[bb14] Sheldrick, G. M. (2008). *Acta Cryst* A**64**, 112–122. 10.1107/S0108767307043930 18156677

